# The association of arterial partial oxygen pressure with mortality in critically ill sepsis patients: a nationwide observational cohort study

**DOI:** 10.1186/s13054-024-04960-w

**Published:** 2024-05-30

**Authors:** Dong-gon Hyun, Jee Hwan Ahn, Jin Won Huh, Sang-Bum Hong, Younsuck Koh, Dong Kyu Oh, Su Yeon Lee, Mi Hyeon Park, Chae-Man Lim, The Korean Sepsis Alliance (KSA) Investigators

**Affiliations:** Department of Pulmonary and Critical Care Medicine, Asan Medical Center, University of Ulsan College of Medicine, 88, Olympic-ro 43-gil, Songpa-gu, Seoul, 05505 Republic of Korea

**Keywords:** Oxygen, Hyperoxia, Hypoxia, Sepsis, Intensive care medicine

## Abstract

**Background:**

Although several trials were conducted to optimize the oxygenation range in intensive care unit (ICU) patients, no studies have yet reached a universal recommendation on the optimal a partial pressure of oxygen in arterial blood (PaO_2_) range in patients with sepsis. Our aim was to evaluate whether a relatively high arterial oxygen tension is associated with longer survival in sepsis patients compared with conservative arterial oxygen tension.

**Methods:**

From the Korean Sepsis Alliance nationwide registry, patients treated with liberal PaO_2_ (PaO_2_ ≥ 80 mm Hg) were 1:1 matched with those treated with conservative PaO_2_ (PaO_2_ < 80 mm Hg) over the first three days after ICU admission according to the propensity score. The primary outcome was 28-day mortality.

**Results:**

The median values of PaO_2_ over the first three ICU days in 1211 liberal and 1211 conservative PaO_2_ groups were, respectively, 107.2 (92.0–134.0) and 84.4 (71.2–112.0) in day 1110.0 (93.4–132.0) and 80.0 (71.0–100.0) in day 2, and 106.0 (91.9–127.4) and 78.0 (69.0–94.5) in day 3 (all *p*-values < 0.001). The liberal PaO_2_ group showed a lower likelihood of death at day 28 (14.9%; hazard ratio [HR], 0.79; 95% confidence interval [CI] 0.65–0.96; *p*-value = 0.017). ICU (HR, 0.80; 95% CI 0.67–0.96; *p*-value = 0.019) and hospital mortalities (HR, 0.84; 95% CI 0.73–0.97; *p*-value = 0.020) were lower in the liberal PaO_2_ group. On ICU days 2 (*p*-value = 0.007) and 3 (*p*-value < 0.001), but not ICU day 1, hyperoxia was associated with better prognosis compared with conservative oxygenation., with the lowest 28-day mortality, especially at PaO_2_ of around 100 mm Hg.

**Conclusions:**

In critically ill patients with sepsis, higher PaO_2_ (≥ 80 mm Hg) during the first three ICU days was associated with a lower 28-day mortality compared with conservative PaO_2_.

**Supplementary Information:**

The online version contains supplementary material available at 10.1186/s13054-024-04960-w.

## Introduction

Sepsis is a life-threatening condition with organ dysfunction caused by impaired oxygen delivery and utilization by cells. Oxygen is often administered to patients with sepsis in an intensive care unit (ICU), especially to those with sepsis-induced hypoxemic respiratory failure [1]. In modern medicine, the conservative goal of oxygen treatment is to maintain a partial pressure of oxygen in arterial blood (PaO_2_) around 60 mm Hg, where > 90% of hemoglobin is saturated [2]. Although this target appears reasonable for patients with preserved oxygen delivery and utilization in tissues, it might not apply to patients with sepsis in which macro and/or micro oxygen transport and cellular utilization are abnormal [3]. In this context, the question arises as to whether the traditional oxygenation strategy, solely focused on arterial blood, is appropriate for sepsis-associated hypoxia, which affects organs and tissues throughout the body [4]. Furthermore, whether a PaO_2_ of 60 mm Hg in the systemic arterial system is sufficient to provide adequate oxygen to organs with dual blood supply, such as the liver (60% by the portal vein) and lung (entirely perfused by venous blood), which often become dysfunctional in sepsis, remains unclear [5].

After a suggestion of a U-shaped relationship between PaO_2_ and mortality in an observational study, several trials have been conducted to optimize the oxygenation range in ICU patients [6–11]. However, despite these numerous studies, studies have not yet reached a universal recommendation on the optimal PaO_2_ range in sepsis treatment [12]. In a previous study of patients with septic shock, those who received mechanical ventilation with a fraction of inspired oxygen (FiO_2_) set at 100% during the first ICU had a higher tendency of mortality than those who had a conventional PaO_2_ target [13]. However, a study focusing on the oxygen target in patients with sepsis has recently suggested that a higher-than-usual oxygen target might lead to a better prognosis [14]. Another recent research found a trend toward higher mortality in patients treated with low oxygen targets (PaO_2_ 55–80 mm Hg) compared to a high oxygenation strategy (PaO_2_ 110–150 mm Hg) [15]. We hypothesized that higher PaO_2_ (≥ 80 mm Hg) is beneficial in critically ill patients with sepsis. Therefore, we aimed to evaluate the effects of a higher oxygenation range on mortality in patients with sepsis compared with conservative therapy to oxygenation.

## Methods

### Study design and patients

This study was conducted based on an ongoing nationwide observational cohort (the Korean Sepsis Alliance registry, KSA), which prospectively collected data on 13,827 patients with sepsis from 15 hospitals in South Korea between September 2019 and December 2022. The registry information, such as inclusion criteria, was introduced in previous studies [16, 17]. All patients from the registry aged ≥ 19 years who were admitted to the ICU for sepsis treatment were included. The exclusion criterion was no data on PaO_2_ over the first three ICU days due to missed data or < 3 days of ICU stay.

### Data collection and oxygenation range

Data recorded in an electronic case report form from the KSA registry were collected, including age, sex, comorbidity, sepsis type, sequential organ failure assessment (SOFA) score, infection site, laboratory finding, sepsis treatment, and microbiology. Comorbidity was identified based on definitions provided by the previous study [1]. We categorized sepsis types into two groups: community-acquired sepsis for a patient who was admitted to the ICU through the emergency room or hospital-acquired sepsis for a patient who was screened by the rapid response team and admitted to the ICU from a ward. SOFA was calculated to evaluate the illness severity at sepsis diagnosis and over the first three ICU days. The infection site was classified as follows: pulmonary, abdominal, urinary, and others. We defined ICU day 1 as the time from ICU admission to the first midnight, ICU day 2 as the next 24 h from the first midnight, and ICU day 3 as the time from the second midnight to the third midnight [16].

The values of PaO_2_ over the first three days of ICU admission were collected. When multiple arterial blood gas analysis was performed, the lowest result regarding PaO_2_ was recorded. Based on the PaO_2_ value from arterial blood gas analysis, patients who maintained a PaO_2_ ≥ 80 mm Hg during the first three days in the ICU were assigned to the liberal PaO_2_ group, while the remaining were included in the conservative PaO_2_ group.

### Propensity score matching and outcomes

Propensity score matching was performed to achieve balance in covariates between liberal and conservative PaO_2_ groups in the entire cohort. The propensity score for the high oxygenation range (PaO_2_ ≥ 80 mm Hg) was estimated using a multivariable logistic regression model with the following covariates: sex, age, comorbidities, sepsis type, initial SOFA score, site of infection, adjunct interventions for sepsis treatment, presence of microbiologic pathogens, total SOFA score and respiratory SOFA score on ICU day 1; and organ support including mechanical ventilation, continuous renal replacement therapy (RRT), vasopressors, and extracorporeal membrane oxygenation on ICU day 1. Patients in the liberal PaO_2_ group were 1:1 matched to those in the conservative PaO_2_ group according to the propensity score with a 1:1 nearest-neighbor algorithm without replacement and with a caliper width of 0.1. Primary and secondary outcomes were assessed in these matched cohorts.

The primary outcome was 28-day mortality after ICU admission. Secondary outcomes comprised ICU mortality, hospital mortality, and 90-day mortality. Additionally, we compared newly onset organ failure during ICU between the two groups, including new-onset invasive ventilation, RRT, arrhythmia, and cardiopulmonary resuscitation. ICU length of stay (LOS) from ICU admission to ICU discharge and hospital LOS from hospital admission to hospital discharge were also measured as secondary outcomes.

### Statistical analysis

Data were shown in numbers and proportions for categorical variables and means ± standard deviations or medians (interquartile range [IQR]) for continuous variables with a normal or non-normal distribution, respectively. Differences for categorical variables were assessed using a chi-squared test. In the propensity score-matched cohort, absolute standardized mean differences (SMDs) were calculated to evaluate the imbalance between the groups before and after matching. The SMD values ≤ 0.1 indicated a lack of any meaningful imbalance. Additionally, groups were compared using a linear mixed model for continuous variables. For 28-day mortality, survival curves were calculated using the Kaplan-Meier method. The hazard ratio (HR) was estimated using the Cox-proportional hazard regression model to compare primary and secondary outcomes. The proportional hazards assumption was evaluated by an inspection of Schoenfeld residuals. The results were presented as an HR with a 95% confidence interval (CI). We also assessed the primary outcome in prespecified subgroups to investigate the relationship between high oxygenation range and heterogenous population in a post hoc analysis. Moreover, discharge from the ICU on day 28 was evaluated via competing-risks regression based on a clustered Fine and Gray’s proportional subhazards model. Death before day 28 was considered the competing event. This analysis provided sub-hazard ratios and 95% CIs. Two-sided *P* values < 0.05 were considered significant. All analyses were performed using R software version 4.1.2 (R Core Team).

## Results

### Patients

**Fig. 1:**
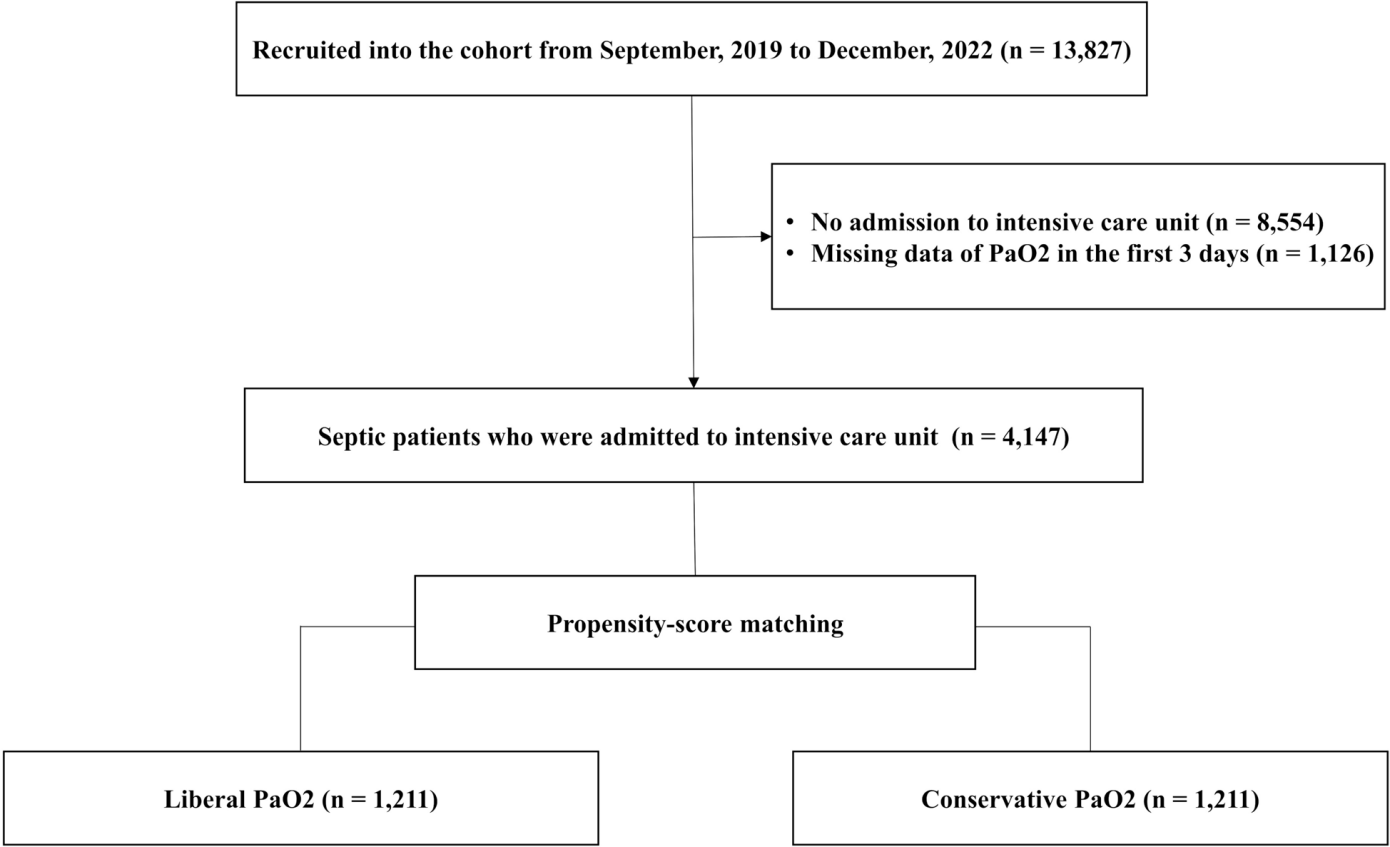
Flow chart of analysis population. PaO_2_, Partial Pressure of Oxygen in Arterial Blood

Of 13,827 patients in the KSA registry, 4147 patients were included in this study (Fig. 1). The baseline characteristics of the entire cohort were similar except for age, sepsis type, infection site, C-reactive protein, mechanical ventilation, and microbiology (Additional file 1: Table S1). After 1:1 propensity score matching that assigned 1211 patients to the liberal PaO_2_ group and 1211 patients to the conservative PaO_2_ group, the differences in baseline characteristics were well-balanced in the matched cohort with the SMD ≤ 10% (Table 1 and Additional file 1: Fig. S1).

**Table 1:** Baseline characteristics of the matched cohort

Characteristic	Conservative PaO_2_ (*n* = 1211)	Liberal PaO_2_ (*n* = 1211)	SMD
Female, n (%)	487 (40.2)	482 (39.8)	0.008
Age, yr, median [IQR]	73.0 [63.0–81.0]	73.0 [63.0–81.0]	0.002
Comorbidities, n (%)			
Cardiac	238 (19.7)	245 (20.2)	0.014
Lung	129 (10.7)	123 (10.2)	0.016
Neurologic	416 (34.4)	414 (34.2)	0.003
Liver	103 (8.5)	113 (9.3)	0.029
Diabetes mellitus	471 (38.9)	466 (38.5)	0.008
Renal disease	168 (13.9)	160 (13.2)	0.019
Connective tissue disease	30 (2.5)	35 (2.9)	0.026
Immunocompromised	48 (4.0)	50 (4.1)	0.008
Hematologic malignancy	74 (6.1)	82 (6.8)	0.027
Solid cancer	358 (29.6)	355 (29.3)	0.005
Sepsis type, n (%)			0.014
Community-acquired sepsis	917 (75.7)	924 (76.3)	
Hospital-acquired sepsis	294 (24.3)	287 (23.7)	
Severity			
SOFA score, median [IQR]	7.0 [5.0–9.0]	7.0 [5.0–9.0]	< 0.001
Site of infection, n (%)			
Respiratory	534 (44.1)	546 (45.1)	0.02
Abdominal	340 (28.1)	332 (27.4)	0.015
Urinary tract	258 (21.3)	254 (21.0)	0.008
Others*	177 (14.6)	178 (14.7)	0.002
Laboratory findings, median [IQR]			
White blood cell count * 10^3^/L	11.5 [6.4–17.6]	11.8 [6.5–18.2]	0.046
C-reactive protein, mg/dL	11.6 [4.8–21.1]	12.2 [5.0–20.8]	0.019
Lactic acid, mmol/L	3.1 [1.9–5.8]	3.2 [1.8–5.6]	0.035
Adjunct interventions, n (%)			
Steroids	263 (21.7)	264 (21.8)	0.002
Mechanical ventilation	586 (48.4)	593 (49.0)	0.012
CRRT	205 (16.9)	207 (17.1)	0.004
ECMO	6 (0.5)	5 (0.4)	0.012
Vasopressors	954 (78.8)	970 (80.1)	0.033
Microbiologic pathogen, n (%)	768 (63.4)	774 (63.9)	0.01
Bacteria	726 (94.5)	722 (93.3)	0.052
Virus	39 (5.1)	40 (5.2)	0.004
Fungus	46 (6.0)	59 (7.6)	0.065

*PaO*_*2*_ Partial pressure of oxygen in arterial blood, *SMD* Standardized mean difference, *IQR* Interquartile range, *SOFA* Sequential organ failure assessment, *CRRT* Continuous renal replacement therapy, *ECMO* Extracorporeal membrane oxygenation.

*Others included skin/soft tissue infection, catheter-associated infection, neurologic infection, and unknown.

### Oxygenation

The median PaO_2_ measured on ICU day 1 was 107.2 mm Hg (IQR, 92.0–134.0) in the liberal PaO_2_ group and 84.4 mm Hg (IQR, 71.2–112.0) in the conservative PaO_2_ group (Additional file 1: Fig. S2). The median PaO_2_ values on ICU days 1–3 were significantly higher (*p*-value < 0.001 on all days) in the liberal PaO_2_ group compared to the conservative group (Table 2). The median FiO_2_ on ICU day 1 in the liberal PaO_2_ group (44.0% [IQR, 32.0–60.0]) was significantly higher compared with the conservative PaO_2_ group (40.0% [IQR, 28.0–60.0]; *p*-value = 0.947), but without significant difference in FiO_2_ on ICU day 2 between the two groups (40.0% [IQR 30.0–50.0] vs. 40.0% [IQR 28.0–50.0]; *p*-value = 0.947). FiO_2_ on ICU day 3 was significantly higher in the conservative PaO_2_ group (36.0% [IQR, 28.0–50.0) than in the liberal PaO_2_ group (35.0 [IQR, 28.0–40.0]; *p*-value = 0.001). No significant difference was observed between the two groups regarding the respiration SOFA score on ICU day 1 (2.0 [IQR, 1.0–2.0] vs. 2.0 [IQR, 1.0–3.0]; *p*-value = 0.717). However, the respiration SOFA score on days 2 (2.0 [IQR, 1.0–3.0] vs. 2.0 [IQR, 1.0–2.0]; *p*-value < 0.001) and 3 (2.0 [IQR, 1.0–3.0] vs. 1.0 [IQR, 1.0–2.0]; *p*-value < 0.001) was higher in the conservative PaO_2_ group. The distribution of patients according to PaO_2_ values for the first three days in the ICU was presented in Additional file 1: Table S2.

**Table 2:** The profile of PaO_2_, FiO_2_, and SOFA over the first three days of ICU admission in the matched cohort

	Conservative PaO_2_ (*n* = 1211)	Liberal PaO_2_ (*n* = 1211)	*p*-value
*ICU day 1, median [IQR]*			
PaO_2_, mm Hg	84.4 [71.2–112.0]	107.2 [92.0–134.0]	< 0.001
FiO_2_, %	40.0 [28.0–60.0]	44.0 [32.0–60.0]	< 0.001
SOFA, total	10.0 [8.0–13.0]	10.0 [8.0–13.0]	0.702
Respiration	2.0 [1.0–2.0]	2.0 [1.0–3.0]	0.717
Coagulation	1.0 [0.0–2.0]	1.0 [0.0–2.0]	0.405
Liver	0.0 [0.0–1.0]	0.0 [0.0–1.0]	0.304
Cardiovascular	4.0 [3.0–4.0]	4.0 [3.0–4.0]	0.955
Central nervous system	2.0 [1.0–3.0]	2.0 [1.0–3.0]	0.031
Renal	1.0 [0.0–2.0]	1.0 [0.0–2.0]	0.574
*ICU day 2, median [IQR]*			
PaO_2_, mm Hg	80.0 [71.0–100.0]	110.0 [93.4–132.0]	< 0.001
FiO_2_, %	40.0 [28.0–50.0]	40.0 [30.0–50.0]	0.947
SOFA, total	11.0 [8.0–13.0]	10.0 [8.0–12.5]	< 0.001
Respiration	2.0 [1.0–3.0]	2.0 [1.0–2.0]	< 0.001
Coagulation	1.0 [0.0–2.0]	1.0 [0.0–2.0]	0.171
Liver	0.0 [0.0–2.0]	0.0 [0.0–1.0]	0.708
Cardiovascular	3.0 [3.0–4.0]	3.0 [2.0–4.0]	0.042
Central nervous system	2.0 [1.0–3.0]	2.0 [1.0–3.0]	0.092
Renal	1.0 [0.0–2.0]	1.0 [0.0–2.0]	0.610
*ICU day 3, median [IQR]*			
PaO_2_, mm Hg	78.0 [69.0–94.5]	106.0 [91.9–127.4]	< 0.001
FiO_2_, %	36.0 [28.0–50.0]	35.0 [28.0–40.0]	0.001
SOFA, total	9.0 [7.0–13.0]	9.0 [6.0–12.0]	< 0.001
Respiration	2.0 [1.0–3.0]	1.0 [1.0–2.0]	< 0.001
Coagulation	2.0 [0.0–3.0]	2.0 [0.0–3.0]	0.259
Liver	0.0 [0.0–2.0]	0.0 [0.0–2.0]	0.287
Cardiovascular	3.0 [0.0–4.0]	3.0 [0.0–4.0]	0.053
Central nervous system	1.0 [1.0–3.0]	1.0 [1.0–3.0]	0.732
Renal	0.0 [0.0–1.0]	0.0 [0.0–1.0]	0.662

*PaO*_*2*_ Partial pressure of oxygen in arterial blood, *FiO*_*2*_ Fraction of inspired oxygen, *SOFA* Sequential organ failure assessment, *ICU* Intensive care unit, *IQR* Interquartile range.

### Outcomes

**Fig. 2:**
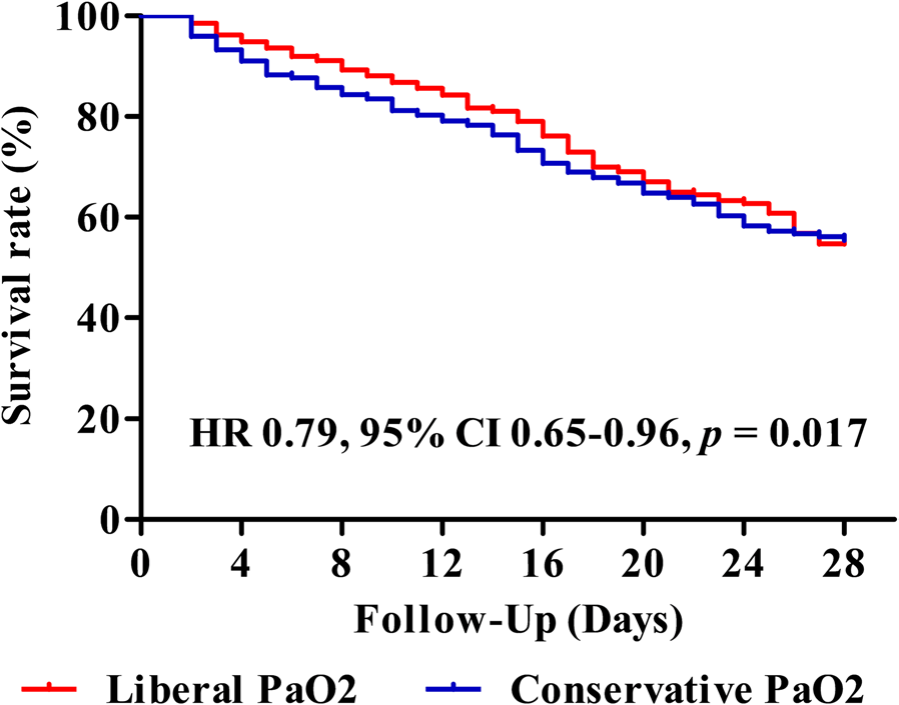
Kaplan-Meier estimates of cumulative probabilities of 28-day survival in propensity-score matched cohort. PaO_2_, Partial pressure of oxygen in arterial blood; HR Hazard ratio; CI Confidence interval

**Fig. 3:**
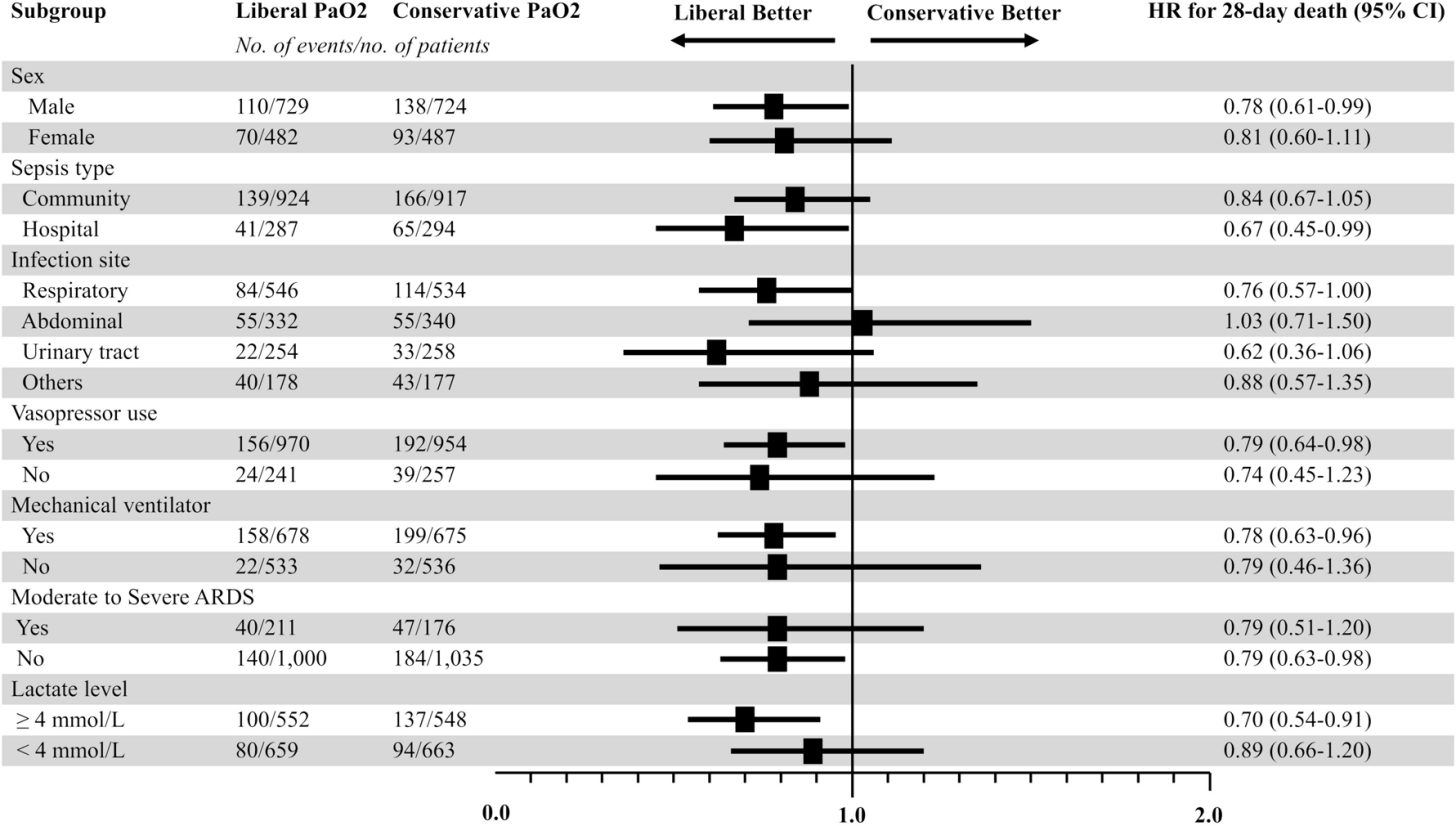
The results of prespecified subgroup analyses of 28-day mortality. PaO_2_, Partial pressure of oxygen in arterial blood; HR, Hazard ratio; CI, Confidence interval; ARDS, Acute respiratory distress syndrome

**Fig. 4:**
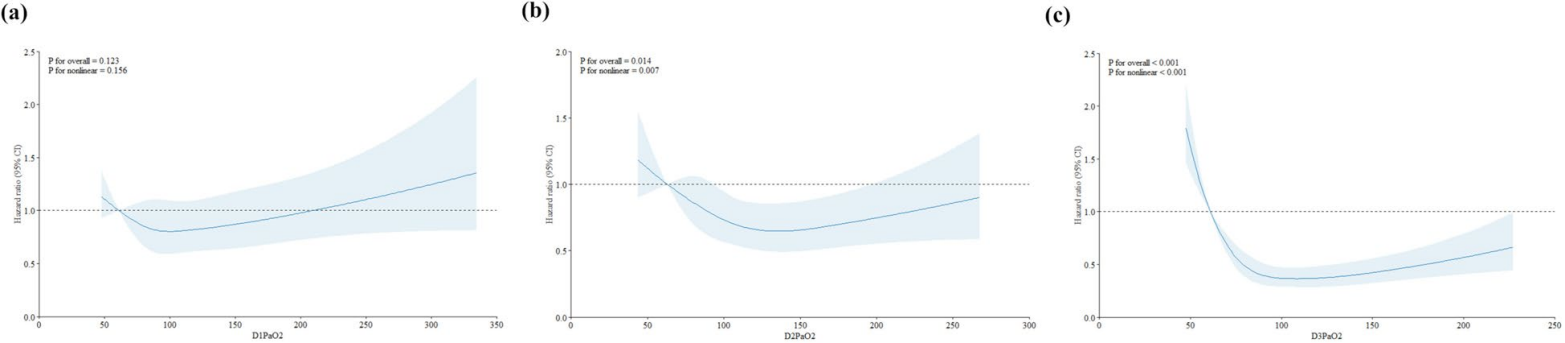
Dose Response Association of PaO_2_ value per ICU day with 28-day mortality. Restricted Cubic Spline Models of Hazard Ratios of PaO_2_ value per ICU day and 28-day Mortality. (A) ICU day 1, (B) ICU day 2, (C) ICU day 3. Knots set at the 5th, 35th, 65th, and 95th percentiles of PaO_2_. Reference is the 5th percentile. Solid lines, hazard ratios; shadow, 95% confidence interval. Model adjusted for age, sex, comorbidities (Lung, Neurology, Liver, Kidney, and Hematology malignancy), infection site, initial sequential organ failure assessment score, lactate level, treatments (steroid and source control), organ support at ICU Day 1 (mechanical ventilation, continuous renal replacement therapy, and vasopressor)

At day 28, mortality was significantly different between the two groups (190 of 1211 patients [14.9%] in the liberal PaO_2_ group and 231 of 1211 patients [19.1%] in the conservative PaO_2_ group). The liberal PaO_2_ group showed a significantly higher probability of survival (HR 0.79, 95% CI 0.65–0.96; *p*-value = 0.017) (Fig. 2). These differences between the two groups were also consistent in early prognosis of 7-day (HR 0.60, 95% CI 0.43–0.83; *p*-value = 0.002) and 14-day mortality (HR 0.73, 95% CI 0.55–0.97; *p*-value = 0.029) (Additional file 1: Fig. S3). In the secondary outcome analysis, similar results were observed between the two groups regarding ICU and hospital mortality (Table 3). Patients in the liberal PaO_2_ group (27.0%) had a lower 90-day mortality than those in the conservative PaO_2_ group (31.3%) but without significant difference (*p*-value = 0.067). Although the incidence of new-onset RRT in the liberal PaO_2_ group (9.2%) was lower than that in the conservative PaO_2_ group (11.6%, *p*-value = 0.062), there were no statistical differences in invasive ventilation, RRT, arrhythmia, and cardiopulmonary resuscitation between the two groups. Although the analysis of ICU and hospital LOS between the two groups did not yield significant differences, the competing risk analysis showed that a higher range of oxygenation was associated with an increased likelihood of ICU discharge at day 28 compared to the conservative oxygenation range, even after adjusting death as a competing event (Additional file 1: Fig. S4). In the subgroup analysis, higher levels of oxygenation were associated with a decreased risk of 28-day mortality in males (HR 0.78, 95% CI 0.61–0.99), patients with hospital-acquired sepsis (HR 0.67, 95% CI 0.45–0.99), those receiving vasopressors (HR 0.79, 95% CI 0.64–0.98), those on a mechanical ventilator (HR 0.78, 95% CI 0.63–0.96), those without moderate to severe acute respiratory distress syndrome (HR 0.79, 95% CI 0.63–0.98), or those with a lactate level ≥ 4 mmol/L (HR 0.70, 95% CI 0.54–0.91) (Fig. 3). Among patients with pulmonary infection, patients in the liberal PaO_2_ group had a low tendency for mortality at day 28 (HR 0.76, 95% CI 0.57–1.00) compared with those in the conservative PaO_2_ group. In the restricted cubic spline model for the dose-response association between PaO_2_ and prognosis, high oxygenation concentration on ICU day 2 (*p* = 0.007) and ICU day 3 (*p* < 0.001) were significantly associated with 28-day mortality after adjustment for covariates (Fig. 4). The risk of 28-day mortality decreased between approximately 100–200 mm Hg of PaO_2_ on ICU Day 2. Additionally, hyperoxemia on ICU Day 3 showed a stronger negative association with 28-day mortality, especially plateauing at PaO_2_ of 100 mm Hg. In analyzing the association between the range of oxygenation and prognosis by the initial ICU date, no differences were observed in all outcomes between the two groups on ICU day 1, whereas differences were found in any outcomes on ICU day 3 (Additional file 1: Table S3). On ICU day 2, a higher oxygenation range was associated with a better prognosis compared to conservative oxygenation regarding mortality only up to days 7 (adjusted HR 0.65, 95% CI 0.49–0.87; *p*-value = 0.004) and 14 (adjusted HR 0.70, 95% CI 0.55–0.90; *p*-value = 0.005).

**Table 3:** Primary and secondary outcomes in the matched cohort

	Conservative PaO_2_	Liberal PaO_2_	HR	95% CI	*p*-value
*Primary outcome*	Reference				
28-day mortality, n (%)	231/1211 (19.1)	180/1211 (14.9)	0.79	0.65–0.96	0.017
*Secondary outcomes*	Reference				
ICU mortality, n (%)	262/1211 (21.6)	202/1211 (16.7)	0.80	0.67–0.96	0.019
Hospital mortality, n (%)	400/1211 (33.0)	344/1211 (28.4)	0.84	0.73–0.97	0.020
90-day mortality, n (%)	379/1211 (31.3)	327/1211 (27.0)	0.87	0.75–1.01	0.067
Invasive ventilation, n (%)	89/1211 (7.3)	85/1211 (7.0)			0.752
Renal replacement therapy, n (%)	140/1211 (11.6)	112/1211 (9.2)			0.062
Arrhythmia, n (%)	182/1211 (15.0)	169/1211 (14.0)			0.453
Cardiopulmonary resuscitation, n (%)	48/1211 (4.0)	39/1211 (3.2)			0.325
ICU LOS, d, median [IQR]	6.0 [3.0–13.0]	6.0 [3.0–12.0]			0.262
Hospital LOS, d, median [IQR]	19.0 [11.0–36.0]	19.0 [12.0–35.0]			0.824

*PaO*_*2*_ Partial pressure of oxygen in arterial blood, *HR* Hazard ratio, *CI* Confidence interval, *ICU* Intensive care unit, *LOS* Length of stay, *IQR* Interquartile range.

## Discussion

Among critically ill adult patients with sepsis in this nationwide cohort study, oxygen supplementation aiming at a PaO_2_ ≥ 80 mm Hg was associated with better outcomes compared with conservative oxygenation therapy. Additionally, hyperoxia on ICU days 2 and 3 was associated with a decreased likelihood of mortality, with the lowest mortality at PaO_2_ of 100 mm Hg. We found apparent differences in the subgroup analysis of 28-day mortality in patients using vasopressors, with lactate level ≥ 4 mmol/L, or without moderate to severe ARDS. Thus, exposure to a higher intensity of oxygen therapy during early ICU days may be associated with reduced mortality in patients with sepsis and these characteristics.

Despite several studies, a controversy about the range of oxygenation in critically ill patients remains, with discrepancies in results from each study [18–20]. For example, the LOCO_2_ trial, including 205 patients with acute respiratory distress syndrome who received liberal (PaO_2_ of 90–105 mm Hg) or conservative (PaO_2_ of 55–70 mm Hg) therapy, showed significantly higher 90-day mortality in the conservative therapy group [8]. Conversely, the HOT-ICU trial, including a larger sample of patients with acute hypoxemic respiratory failure than the LOCO_2_ trial, demonstrated no significant difference in 90-day mortality according to the oxygenation range [9]. The conventional target of PaO_2_ ≥ 60 mm Hg for tissue oxygenation has long been regarded as indisputable [2]. However, a recently conducted study, which utilized a machine learning model to investigate whether the effects of oxygenation targets on outcomes differ based on individual characteristics of patients in the ICU-ROX trial, might provide a solution to this controversy [21]. This study suggested that using individualized oxygenation targets might improve outcomes for critically ill patients receiving mechanical ventilation. For instance, treating septic patients with a high oxygenation target could reduce absolute mortality by 13.0%, which is supported by our study results. Therefore, the varying results of studies on optimal oxygenation do not negate the role of a higher oxygenation target but rather suggest that different diseases or severities of a specific disease may require different oxygenation strategies.

There may be a plausible mechanism of better survival under hyperoxia in patients with sepsis [22]. Besides the low oxygen affinity of erythrocytes, microcirculation during sepsis is characterized by attenuated local oxygen tension gradient, increased capillaries stop flow, reduced functional capillary density, and increased effective tissue volume, altogether leading to decreased oxygen transport to mitochondria by increasing the critical oxygen diffusion distance [23, 24]. However, excess oxygen may help correct deranged cellular metabolic abnormalities in sepsis, resulting in better survival. This effect could be particularly noticeable in tissues supplied with dual blood supply from arterial and venous systems, such as the liver and lungs [25]. Therefore, increased oxygen tension achieved in the circulation, such as higher PaO_2_, may help overcome these sepsis-induced disadvantages of cellular oxygenation [22]. In line with this theory, a secondary analysis of the HOT-ICU trial, involving 2,888 patients with acute hypoxic respiratory failure, suggested a dose-response relationship between norepinephrine dose and increased mortality in those with a lower oxygenation target [26]. In the subgroup analysis of our study, the higher PaO_2_ range compared to the conservative PaO_2_ range was also associated with reduced 28-day mortality in patients using vasopressors or with lactate ≥ 4 mmol/L. Additionally, our results showed that the high oxygenation range on ICU days 2 and 3, but not on ICU day 1, was significantly associated with a lower likelihood of 28-day mortality. Thus, aiming for the high oxygenation range did not have a sufficient effect on the first day of ICU because patients’ macrocirculation, such as mean arterial blood pressure, was usually not yet recovered. However, on ICU days 2 and 3 after the stabilization of macrocirculation, the impact of high oxygenation treatment on prognosis may be more significant.

The strengths of our study include a large sample from the multicenter, nationwide database that might help identify a precise estimate of oxygenation target and enhance generalizability for patients with sepsis in real practice. Nevertheless, several limitations warrant attention. First, the PaO_2_ value might have not properly reflected the actual hyperoxemia status of patients during all days because we could not collect data on the frequency of PaO_2_ analysis and continuously measured PaO_2_. Although the liberal group, which conformed well to the hypothesis of this study, might have reflected the benefits of hyperoxemia because it comprised only patients with a minimum PaO_2_ value over 80 mm Hg for 3 days, this limitation might have attenuated the hyperoxia contrast between the groups. Second, structural limitations in the database compelled us to restrict the comparison period of oxygenation to the first three ICU days. Our aim was to investigate the effect of oxygenation range on prognosis during the early stages of treatment in critically ill patients as long as possible. However, the comparison period was set to the first three ICU days because our database had sequential PaO_2_ values only for the first three ICU days. Additional studies will be needed in the future to determine the appropriate period when the initial oxygenation range has an effect. Third, we excluded patients who died within the first three ICU days because the prognostic effect of the initial oxygenation range could be masked by those deaths. Although this was an appropriate exclusion criterion considering a previous study, it could have induced bias in the results of this study. Fourth, this study was not a randomized trial. Even though the propensity score matching process could balance variables between the groups, potential differences in unmeasured variables might remain. Fifth, additional interventions, except for the PaO_2_ value, especially after ICU day 3, were not controlled due to the nature of the prospectively collected cohort study. Finally, the findings of our study cannot be generalized to patients who received long-term ICU care because our oxygenation range focused on the first three ICU days after sepsis diagnosis.

## Conclusions

In this nationwide observational cohort of sepsis, treatment with relatively higher PaO_2_ was associated with lower 28-day mortality compared to conservative PaO_2_ among critically ill patients with sepsis. Particularly, patients who maintained PaO_2_ ≥ 100 mm Hg on ICU days 2 and 3 showed the lowest 28-day mortality. Additionally, a higher oxygenation range was an independent factor for survival in sepsis with certain conditions. Our study together with a few previous studies indicates that the ‘one size fits all’ oxygenation strategy needs to be re-appraised, especially in sepsis. Future studies on optimal oxygenation in disease need to narrow the subjects to a more homogeneous group of patients.

## Electronic supplementary material

Below is the link to the electronic supplementary material.

Supplementary Material 1

## Data Availability

No datasets were generated or analysed during the current study.

## Abbreviations

ICU: Intensive care unit
PaO_2_: Partial pressure of oxygen in arterial blood
SOFA: Sequential organ failure assessment
CRRT: Continuous renal replacement therapy
LOS: Length of stay
IQR: Interquartile range
SMD: Standardized mean differences
HR: Hazard ratio
CI: Confidence interval
