# Supplementary material for: The association of arterial partial oxygen pressure with mortality in critically ill sepsis patients: a nationwide observational cohort study
Source: Crit Care. 2024 May 30;28:187. doi: 10.1186/s13054-024-04960-w (PMC11140987; doi:10.1186/s13054-024-04960-w)
Supplement: Supplementary file 1 — Supplementary Material 1 [file 13054_2024_4960_MOESM1_ESM.docx]

**Supplemental material**

**The Association of Arterial Partial Oxygen Pressure with Mortality in Critically Ill Sepsis Patients: A nationwide observational cohort study**

Dong-gon Hyun, MD, Jee Hwan Ahn, MD, Jin Won Huh, MD, Ph.D., Sang-Bum Hong, MD, Ph.D., Younsuck Koh, MD, Ph.D., FCCM, Dong Kyu Oh, MD, Su Yeon Lee, MD, Mi Hyeon Park, and Chae-Man Lim, MD, Ph.D., FCCM^*^ on behalf of the Korean Sepsis Alliance (KSA) Investigators


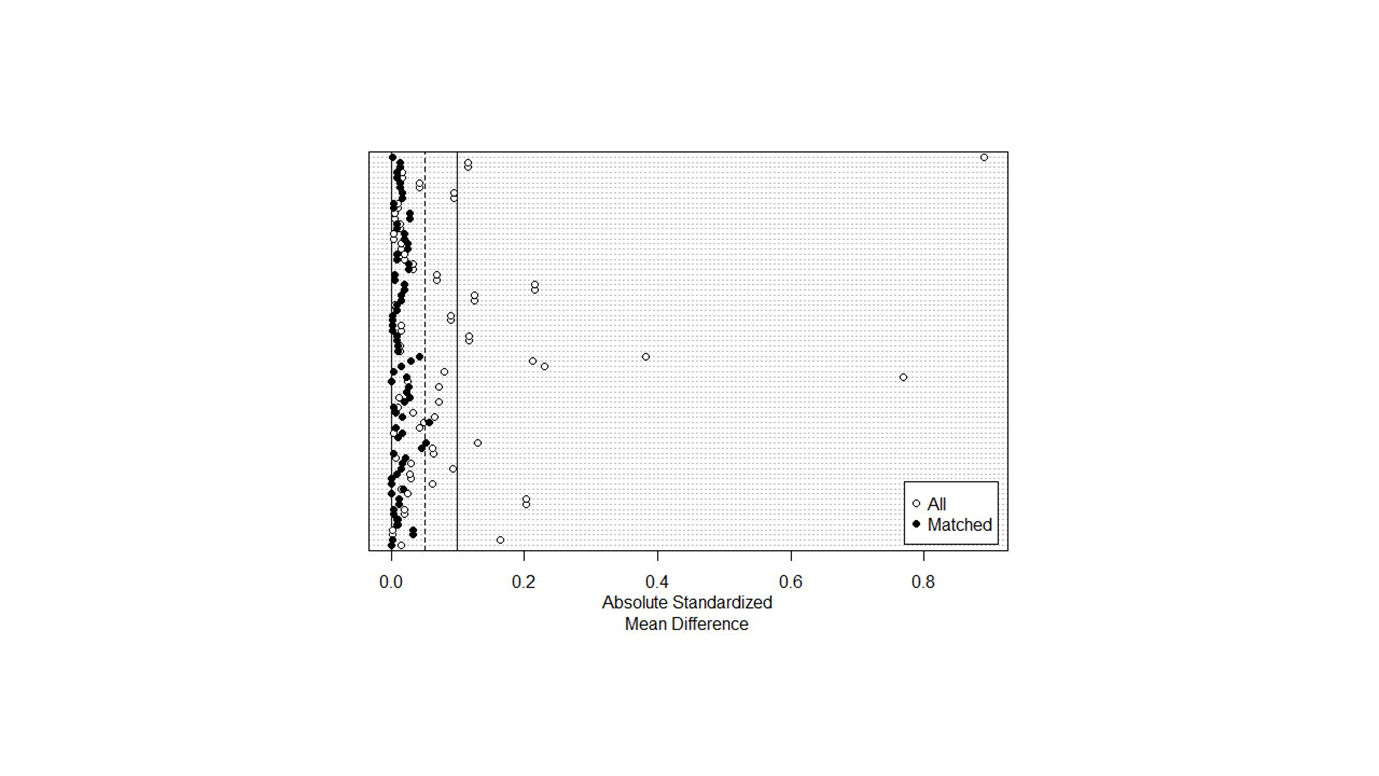


**Fig. S1** Absolute standardized mean differences between liberal and conservative PaO_2_ groups before and after propensity score matching. The *horizontal axis* represents the standardized mean differences, and the *dashed line* indicates the absolute standardized mean difference of 0.1. *Open dots* reflect values prior to matching, and *black dots* reflect values after matching. Matching succeeded in reducing the standardized mean difference within an absolute value of 0.1.


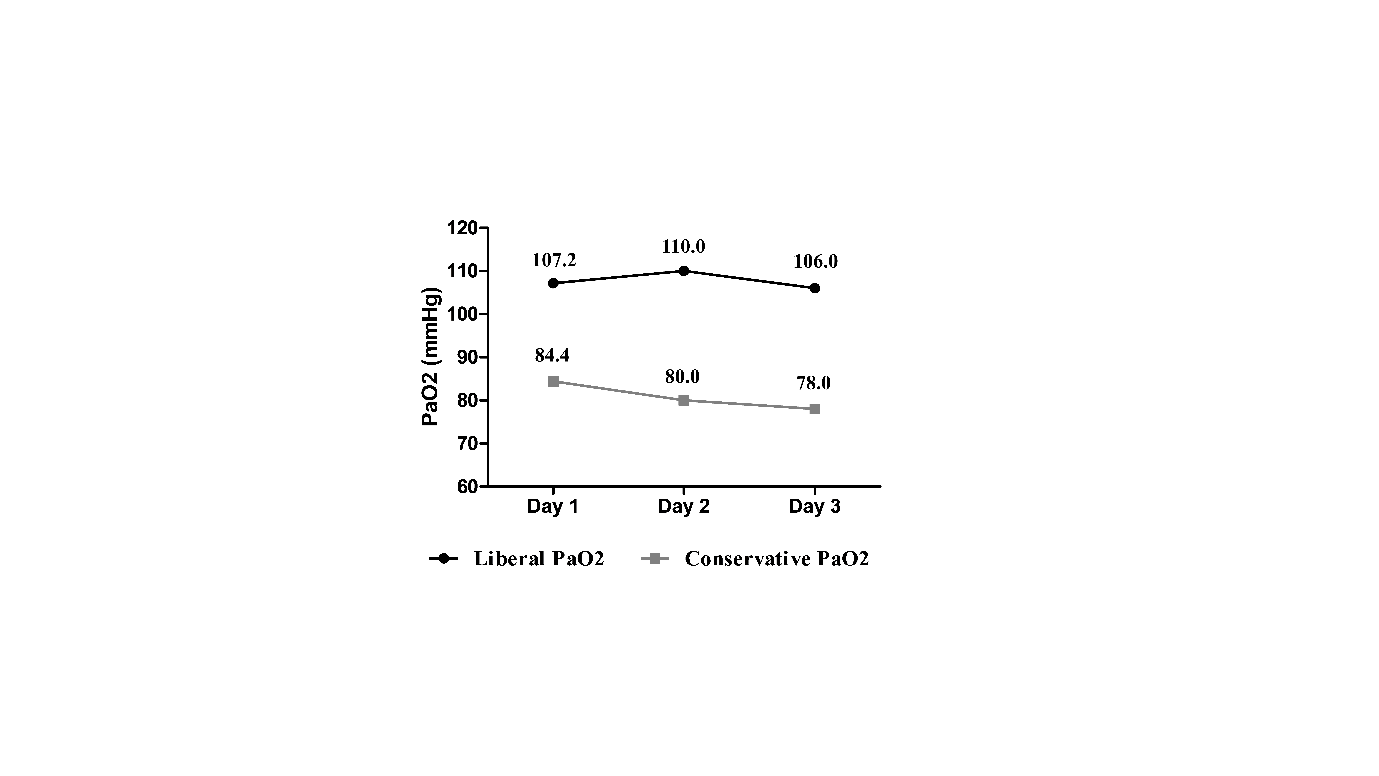


**Fig. S2** The daily median value of the lowest partial pressure of oxygen in arterial blood (PaO_2_) in matched cohorts during the first three days of ICU management in liberal and conservative PaO_2_ groups.


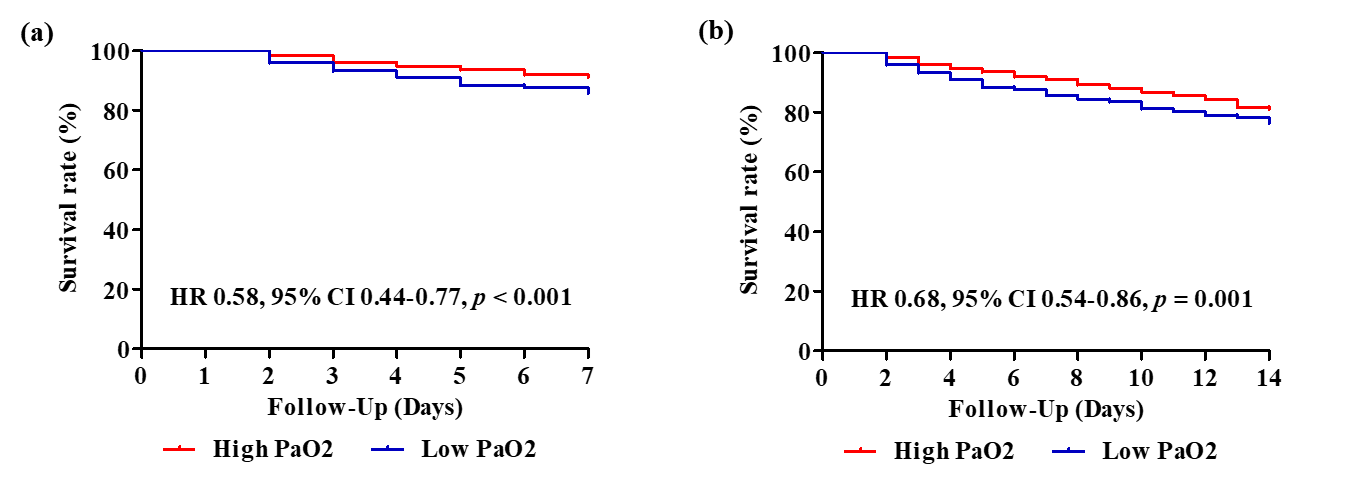


**Fig. S3** Kaplan-Meier analysis of survival censored at 7 days (a) and 14 days (b). PaO2, partial pressure of oxygen in the arterial blood; HR, hazard ratio; CI, confidence interval.


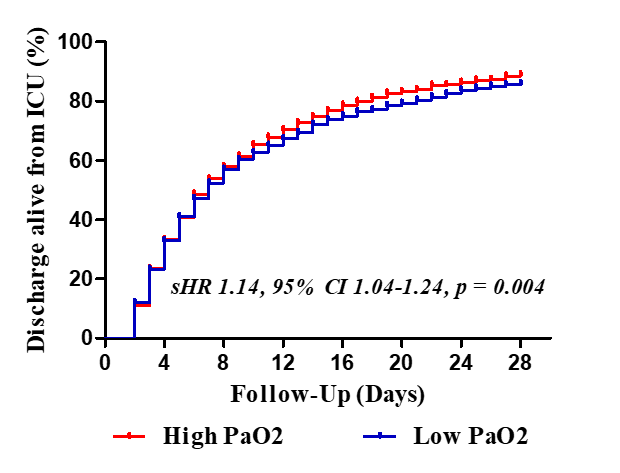


**Fig. S4** Cumulative ICU discharge at day 28 between the two groups. PaO2, partial pressure of oxygen in the arterial blood; sHR, subhazard ratio; CI, confidence interval.

**Table S1** Baseline characteristics of all included patients

| **Characteristic** | **Conservative PaO2 (n = 2,536)** | **Liberal PaO2 (n = 1,611)** | ***p*-value** |
| --- | --- | --- | --- |
| Female, n (%) | 1,022 (40.3) | 662 (41.1) | 0.635 |
| Age, yr, median [IQR] | 74.0 [64.0–82.0] | 72.0 [61.0–80.0] | <0.001 |
| Comorbidities, n (%) |  |  |  |
| Cardiac | 478 (18.9) | 331 (20.6) | 0.192 |
| Lung | 314 (12.4) | 155 (9.6) | 0.007 |
| Neurologic | 891 (35.1) | 558 (34.6) | 0.769 |
| Liver | 232 (9.2) | 145 (9.0) | 0.916 |
| Diabetes mellitus | 946 (37.3) | 611 (37.9) | 0.710 |
| Renal disease | 350 (13.8) | 20 (13.7) | 0.931 |
| Connective tissue disease | 74 (2.9) | 43 (2.7) | 0.707 |
| Immunocompromised | 95 (3.8) | 67 (4.2) | 0.557 |
| Hematologic malignancy | 183 (7.2) | 103 (6.4) | 0.339 |
| Solid cancer | 674 (26.6) | 479 (29.7) | 0.030 |
| Sepsis type, n (%) |  |  | <0.001 |
| Community-acquired sepsis | 2,011 (79.3) | 1,196 (74.2) |  |
| Hospital-acquired sepsis | 525 (20.7) | 415 (25.8) |  |
| Severity |  |  |  |
| SOFA score, median [IQR] | 7.0 [5.0–9.0] | 7.0 [5.0–9.0] | 0.573 |
| Site of infection, n (%) |  |  |  |
| Respiratory | 1,319 (52.0) | 667 (41.4) | <0.001 |
| Abdominal | 612 (24.1) | 481 (29.9) | <0.001 |
| Urinary tract | 516 (20.4) | 324 (20.1) | 0.885 |
| Others* | 323 (12.7) | 258 (16.0) | 0.004 |
| Laboratory findings, median [IQR] | | | |
| White blood cell count * 10^3^/L | 11.5 [6.5–17.1] | 11.9 [6.6–18.0] | 0.163 |
| C-reactive protein, mg/dL | 12.6 [5.4–22.1] | 11.3 [4.5–20.0] | <0.001 |
| Lactic acid, mmol/L | 3.2 [1.9–5.9] | 3.2 [1.8–5.6] | 0.124 |
| Adjunct interventions, n (%) |  |  |  |
| Steroids | 579 (22.8) | 358 (22.2) | 0.675 |
| Mechanical ventilation | 1,123 (44.3) | 876 (54.4) | <0.001 |
| CRRT | 457 (18.0) | 278 (17.3) | 0.558 |
| ECMO | 18 (0.7) | 13 (0.8) | 0.866 |
| Vasopressors | 2,009 (79.2) | 1,275 (79.1) | 0.985 |
| Microbiologic pathogen, n (%) | 1,611 (63.5) | 1,034 (64.2) | 0.691 |
| Bacteria | 1,510 (93.7) | 967 (93.5) | 0.893 |
| Virus | 94 (5.8) | 53 (5.1) | 0.490 |
| Fungus | 96 (6.0) | 83 (8.0) | 0.047 |

*Others included skin/soft tissue infection, catheter-associated infection, neurologic infection, and unknown.

*PaO_2,_* partial pressure of oxygen in arterial blood; *IQR,* interquartile range; *SOFA,* sequential organ failure assessment; *CRRT,* continuous renal replacement therapy; *ECMO,* extracorporeal membrane oxygenation; *MDR,* multi-drug resistance

**Table S2** Distribution of patients according to PaO2 values for the first three days in the ICU

|  | **Conservative PaO2 (n = 1,211)** | **Liberal PaO2 (n = 1,211)** | ***p*-value** |
| --- | --- | --- | --- |
| PaO2 at ICU Day 1 |  |  | < 0.001 |
| ≥ 80 mm Hg | 556 (45.9) | 0 (0.0) |  |
| < 80 mm Hg | 655 (54.1) | 1,211 (100.0) |  |
| PaO2 at ICU Day 2 |  |  | < 0.001 |
| ≥ 80 mm Hg | 606 (50.0) | 0 (0.0) |  |
| < 80 mm Hg | 605 (49.9) | 1,211 (100.0) |  |
| PaO2 at ICU Day 3 |  |  | < 0.001 |
| ≥ 80 mm Hg | 694 (57.3) | 0 (0.0) |  |
| < 80 mm Hg | 517 (42.7) | 1,211 (100.0) |  |

*PaO_2,_* partial pressure of oxygen in arterial blood; ICU, intensive care unit.

**Table S3** Subgroup analysis of clinical outcomes based on PaO_2_ by ICU date

|  | **Conservative PaO2** | **Liberal PaO2** | **HR (95% CI)*** | ***p*-value** |
| --- | --- | --- | --- | --- |
| ICU Day 1, n (%) | n = 556 | n = 1,866 |  |  |
| Death at 7 days | 57 (10.3) | 161 (8.6) | 0.78 (0.58-1.06) | 0.111 |
| Death at 14 days | 74 (13.3) | 229 (12.3) | 0.84 (0.65-1.10) | 0.205 |
| Death at 28 days | 97 (17.4) | 314 (16.8) | 0.87 (0.69-1.09) | 0.232 |
|  | | | | |
| ICU Day 2, n (%) | n = 606 | n =1,816 |  |  |
| Death at 7 days | 71 (11.7) | 147 (8.1) | 0.65 (0.49-0.87) | 0.004 |
| Death at 14 days | 96 (15.8) | 207 (11.4) | 0.70 (0.55-0.90) | 0.005 |
| Death at 28 days | 123 (20.3) | 288 (15.9) | 0.82 (0.66-1.02) | 0.068 |
|  | | | | |
| ICU Day 3, n (%) | n = 694 | n = 1,728 |  |  |
| Death at 7 days | 99 (14.3) | 119 (6.9) | 0.45 (0.35-0.60) | < 0.001 |
| Death at 14 days | 124 (17.9) | 179 (10.4) | 0.54 (0.42-0.68) | < 0.001 |
| Death at 28 days | 156 (22.5) | 255 (14.8) | 0.60 (0.49-0.73) | < 0.001 |

*PaO_2,_* partial pressure of oxygen in arterial blood; HR, hazards ratio; CI, confidence interval; ICU, intensive care unit.

* Adjusted for age, sex, comorbidities (Lung, Neurology, Liver, Kidney, and Hematology malignancy), infection site, initial sequential organ failure assessment score, lactate level, treatments (steroid and source control), organ support at ICU Day 1 (mechanical ventilation, continuous renal replacement therapy, and vasopressor).

**Table S4** Cox proportional hazards model of factors associated with mortality at 28 days.

|  | **Univariable analysis** | | |  |
| --- | --- | --- | --- | --- |
| **Variable** | **OR** | **95% CI** | ***p-*value** |  |
| Female | 1.12 | 0.92-1.36 | 0.266 |  |
| Age, yr, | 1.00 | 1.00-1.01 | 0.410 |  |
| Comorbidities |  |  |  |  |
| Cardiac | 1.07 | 0.84-1.35 | 0.580 |  |
| Lung | 1.32 | 0.99-1.75 | 0.056 |  |
| Neurologic | 0.78 | 0.63-0.96 | 0.020 |  |
| Liver | 1.41 | 1.04-1.90 | 0.028 |  |
| Diabetes mellitus | 1.08 | 0.89-1.32 | 0.431 |  |
| Renal disease | 1.34 | 1.04-1.72 | 0.022 |  |
| Connective tissue disease | 1.16 | 0.68-1.98 | 0.576 |  |
| Immunocompromised | 0.99 | 0.64-1.53 | 0.949 |  |
| Hematologic malignancy | 1.69 | 1.24-2.31 | 0.001 |  |
| Solid cancer | 1.05 | 0.85-1.30 | 0.632 |  |
| Sepsis type |  |  |  |  |
| Hospital-acquired sepsis | 0.98 | 0.79-1.23 | 0.886 |  |
| Severity |  |  |  |  |
| SOFA score | 1.08 | 1.05-1.11 | <0.001 |  |
| Site of infection |  |  |  |  |
| Respiratory | 0.83 | 0.69-1.01 | 0.067 |  |
| Abdominal | 1.15 | 0.92-1.43 | 0.213 |  |
| Urinary tract | 0.78 | 0.58-1.03 | 0.083 |  |
| Others* | 1.38 | 1.08-1.75 | 0.009 |  |
| Laboratory findings |  |  |  |  |
| White blood cell count * 10^3^/L | 1.00 | 0.99-1.01 | 0.925 |  |
| C-reactive protein, mg/dL | 0.99 | 0.99-1.00 | 0.217 |  |
| Lactic acid, mmol/L | 1.07 | 1.05-1.10 | <0.001 |  |
| Adjunct interventions |  |  |  |  |
| Steroids | 1.23 | 0.99-1.53 | 0.056 |  |
| Mechanical ventilation | 1.30 | 1.05-1.61 | 0.016 |  |
| CRRT | 1.64 | 1.32-2.03 | <0.001 |  |
| ECMO | 0.94 | 0.35-2.52 | 0.898 |  |
| Vasopressors | 1.40 | 1.07-1.83 | 0.014 |  |
| Microbiologic pathogen |  |  |  |  |
| Bacteria | 0.97 | 0.61-1.53 | 0.891 |  |
| Virus | 1.14 | 0.73-1.78 | 0.563 |  |
| Fungus | 1.11 | 0.72-1.70 | 0.638 |  |
| PaO2 value |  |  |  |  |
| PaO2 at ICU Day 1 | 1.00 | 1.00-1.00 | 0.013 |  |
| PaO2 at ICU Day 2 | 1.00 | 1.00-1.00 | 0.500 |  |
| PaO2 at ICU Day 3 | 1.00 | 0.99-1.00 | 0.006 |  |

*Others included skin/soft tissue infection, catheter-associated infection, neurologic infection, and unknown.

PaO_2_, partial pressure of oxygen in arterial blood; SOFA, sequential organ failure assessment; CRRT, continuous renal replacement therapy; ECMO, extracorporeal membrane oxygenation.
